# Expression profiling of *KRAS* and *NOXA* genes as prospective biomarkers in ovarian carcinoma

**DOI:** 10.1038/s41598-025-17650-6

**Published:** 2025-09-05

**Authors:** Kholoud Abdelnaeem, Reham Mohammed Dawood, Basma E. Fotouh, Abeer Ismail, Mohga S. Abdalla, Shimaa S. Ramadan

**Affiliations:** 1 Molecular Biotechnology Sector, Chemistry Department, Faculty of Science, Helwan University, Cairo, Egypt; 2 Microbial Biotechnology Department, Biotechnology Research Institute, National Research Centre, EL Bohouth St. Dokki, P.O. 12622, Giza, Egypt; 3 Department of Clinical Pathology, National Cancer Institute, Cairo University, Cairo, Egypt; 4 Biochemistry Sector, Chemistry Department, Faculty of Science, Helwan University, Cairo, 11795 Egypt

**Keywords:** Ovarian cancer, Gene expression, *KRAS*, *NOXA*, Disease progression, Biotechnology, Molecular biology

## Abstract

**Background:**

Ovarian cancer (OC) is a leading cause of cancer deaths in women. Comprehensive molecular studies are required to understand OC pathogenesis. *KRAS* and *NOXA* genes are involved in tumorigenesis and disease progression. *KRAS* promotes tumor growth, while *NOXA* triggers the apoptotic signaling pathway.

**Methods:**

Fifty-six ovarian cancer patients and twenty healthy controls were enrolled in the study. Gene expression profiling was performed utilizing the qRT-PCR assay. Then, the proteomic levels of *KRAS* and *NOXA* genes were assessed by the ELISA technique.

**Results:**

The results showed that *KRAS* gene expression was significantly increased in OC patients compared to controls (*p* < 0.0001). Additionally, *KRAS* overexpression was correlated significantly with more aggressive characteristics, including advanced stage, positive lymph invasion, and metastatic status (*p* = 0.04, 0.05, and 0.02, respectively). In contrast, *NOXA* expression was downregulated in OC patients compared to controls (*p* = 0.001), and this reduction was more pronounced in patients with more aggressive characteristics (*p* = 0.001, 0.01, 0.0008, 0.02, respectively). At the transcriptomic level, *KRAS* concentration was higher among the patient group (*p* = 0.03) and correlated with aggressive tumor features (*p* = < 0.0001, 0.001, and 0.03, respectively). Interestingly, no significant changes were detected in *NOXA* protein levels concerning ovarian cancer development and progression. These findings were further confirmed by receiver operating characteristic (ROC) curve analysis, validating the genetic and transcriptomic results.

**Conclusion:**

The differential expression of *KRAS* and *NOXA* genes holds promise as potential biomarkers for ovarian cancer development and progression. Specifically, *KRAS* transcriptomic levels serve as a reliable discriminator of ovarian cancer progression. In contrast, *NOXA* protein expression warrants further investigation to elucidate its role in the progression and pathophysiology of ovarian cancer.

## Introduction

Ovarian cancer (OC) represented the most lethal malignancy of all gynecological cancers and was distinguished by the worst prognosis with the highest mortality rate. Globally, it is the fifth most common cancer among females, accounting for 3.7% of all cases and 4.7% of cancer-related deaths. By 2040, more than 445,721 cases are expected to be diagnosed with ovarian cancer, and its fatality rate is expected to increase dramatically^1^. In Egypt, the National Cancer Registry reported that ovarian cancer constitutes 2.2% of all cancer cases and 4.4% of newly diagnosed cancers among women. Besides, in 2022, 3,070 cases of ovarian cancer were reported, highlighting its significant impact on women’s health^2^.

Ovarian cancer is characterized by uncontrolled cell growth within the ovaries, with more than 85% of patients having epithelial ovarian cancers (EOCs). The specific etiology of ovarian cancer is unknown, although various variables might raise the chance of developing the malignancy. Lifestyle factors, hormonal and environmental influences have all been implicated. Moreover, getting older, menopausal status, and a family history of breast cancer serve as notable risk factors^3^. Additionally, critical genetic elements, such as mutations and dysregulation of expression, represent significant contributors to the development of ovarian cancer^4^. All the aforementioned factors significantly shape the distinct patterns and trends related to ovarian cancer incidences and fatalities globally.

This disease is intricate and multifaceted, owing to its heterogeneous nature, which encompasses a variety of histological subtypes, each exhibiting unique biological behaviors, complex molecular features, and responses to treatment^5^. Moreover, the lack of distinct symptoms and efficient, trustworthy biomarkers leads to a diagnosis of the disease at end-stage. It was reported that more than 75% of patients are diagnosed at more advanced stages (III or IV) with metastasis, which decreases the 5-year survival rate (< 30%)^6^.

Besides, the cornerstone treatment strategies, including surgical resection and chemotherapy present several limits, such as chemo-resistance, tumor recurrence, and a lower chance of curability^7^. Notably, it has been reported that advancement, invasion of ovarian cancer, and treatment response are characterized by the acquisition of multiple genetic mutations and alterations in gene expression 8^10^. At present, early identification approaches, therapies, and monitoring to increase survival and quality of life are still inadequate^11^. Consequently, a comprehensive molecular understanding of (OC) pathogenesis and the underlying mechanisms of its progression is vital for effectively managing this lethal and highly metastatic disease.

Analyzing gene expression profiles related to tumorigenesis represents a significant research approach that integrates genetic and molecular transcription information to target genes that are dysregulated in neoplasm development. Consequently, it contributes to early detection and offers insights into prognosis and tumor invasiveness. Studies have identified various prognostic molecular biomarkers for ovarian cancer by elucidating alterations in the expression levels of diverse genes, including *Notch*,* FOXM1*,* Nuclear β-catenin*,* E-cadherin*, *PTEN*,* AURKA*,* CD24*,* PGR*, and mitochondrial genes^9,12,13^. Nevertheless, the search for effective screening remains an ongoing challenge, as this is a crucial medical demand that hasn’t yet been fully addressed. In this context, there is a growing interest in exploring genomics and proteomics to understand how they regulate various signaling pathways.

The *KRAS* gene (Kirsten rat sarcoma viral oncogene homolog) has an oncogenic function and belongs to a group of small GTP-binding proteins, known as RAS-like GTPases. It is located on chromosome 12p and encodes a small GTPase transductor protein called *KRAS.* Notably, *KRAS* signaling orchestrates a diverse array of cellular signaling pathways, such as mitogen-activated protein kinase *(MAPK)* and P*I3K/AKT.* These signals are pivotal in processes such as cellular growth, transformation, and angiogenesis, ultimately driving tumorigenesis and progression^14^.

Irreversible changes in cell genetics are a major factor in altering gene expression and protein function that regulate cellular growth and differentiation, ultimately leading to tumorigenesis^15,16^. Notably, gene set enrichment analysis (GSEA) has identified *KRAS* as one of the hub genes and essential hallmarks of ovarian neoplastic state development^17^. Additionally, elevated levels of *KRAS* expression are associated with unfavorable patient outcomes in different types of cancer, suggesting its potential as a prognostic biomarker^18^.

Phorbol-12-myristate-13-acetate-induced protein 1 *(PAMIP1) (NOXA)*, a pro-apoptotic member of the Bcl-2 family that is located on chromosome 18. It is involved in cancer development and progression by regulating apoptosis and influencing therapeutic responses. *NOXA* facilitates apoptosis indirectly through binding anti-apoptotic proteins (Bcl-2 family protein myeloid cell leukemia-1(Mcl-1), which is overexpressed in many cancers, including ovarian cancer, leading to progression and poor prognosis^19^.This ultimately promotes pro-apoptotic Bax/Bak activation, mitochondrial outer membrane permeabilization (MOMP), and triggers the release of cytochrome C along with caspase activation and the execution of apoptosis^20^.

The expression levels of *NOXA* differ significantly among cancer types^21^. In some cancers, *NOXA* exhibits tumor-suppressive properties by promoting apoptosis in response to anticancer drugs. Conversely, its aberrant regulation may contribute to tumorigenesis in other malignancies and be associated with aggressive phenotypes and unfavourable outcomes^22,23^. Accordingly, gaining a comprehensive understanding of the multifaceted role of *NOXA* in carcinogenesis and invasion provides valuable insights about enhancing apoptosis in cancer cells, inhibiting tumor invasion, and enhancing overall quality of life.

The current study aims to clarify the role of the *NOXA and KRAS* genes that could be involved in OC development and progression. Therefore, the transcriptomic and protein expression levels of both genes were evaluated in OC patients in comparison to healthy individuals.

## Results

### Socio-demographic, pathological, and clinical profile of enrolled participants

The current study involved 76 female participants, divided into two groups: The control group (*n* = 20) and the ovarian cancer patients (*n* = 56). The demographics, clinical, and pathological data of all individuals were recapitulated in Table 1. The mean age of the patients and control group was (53.8 ± 11 and 35.9 ± 12), respectively, and 73.2% of patients were ≥ 45 years old. The most common histological subtype was serous carcinoma, constituting 67.9% of cases, whereas non-serous subtypes (endometrioid carcinoma, mucinous carcinoma, and clear cell carcinoma) were identified in 32.1%. Data showed that postmenopausal status was more common among ovarian cancer patients. The presence of a previous cancer family history was more prevalent in the patient group compared to the controls. In the context of ovarian complaints,, 42.9% of patients had severe abdominal pain only, and 26.8% had severe abdominal pain with vaginal bleeding.The rest of the patients (30.3%) had different complaints such as weight loss, ascites, and lower back pain.

**Table 1:** Comparision of Demographic and Clinicopathological characteristics between patients with ovarian carcinoma and control group.

Variables	Controls (*n* = 20)	Malignant OC patients (*n* = 56)
Age (Years)	35.9 ± 12	53.8 ± 11
*Age group (year)*		
< 45	13(65.0%)	15 (26.8%)
≥ 45	7(35.0%)	41 (73.2%)
*Family history*		
Negative	17(85.0%)	34 (60.7%)
Positive	3 (15.0%)	19 (33.9%)
*Menopausal status*		
Postmenopause	5 (25.0%)	35 (62.5%)
Premenopause	15 (75.0%)	21 (37.5%)
*Tumor Side*		
Unilateral		35 (62.5%)
Bilateral		21 (37.5%)
*Histological types*		
Serous		38 (67.9%)
Non-serous		18 (32.1%)
*FIGO stage*		
I		10 (17.9) %
II		18 (32.1%)
III		14 (25.0%)
IV		14 (25.0%)
*Tumor grade*		
I		24 (42.9%)
II		32(57.1%)
*Lymph nodes Invasion*		
N0		17(30.4%)
N1		13(23.2%)
N2		9 (16.1%)
N3		17(30.3%)
*Tumor size*		
T1		16 (28.5%)
T2		13 (23.2%)
T3		20 (35.7%)
T4		7 (12.5%)
*Metastatic status*		
M0		42 (75.0%)
M1		14 (25.0%)
*Biomarkers*		
CA125(U/ml)		733 ± 11184
HE4(pmol/L)		494 ± 416
*Complaints*		
Severe Abdominal pain		24 (42.9%)
Severe Abdominal pain, Vaginal bleeding		15 (26.8%)
Others		17 (30.3%)

The pathological characteristics of OC showed that 62.5% of patients had ovarian carcinoma in a unilateral site. Besides, the data revealed that 57.1% of patients had moderately differentiated tumors (grade II). Regarding the tumor size, 35.7% of patients had a big tumor size (T3). Moreover, 30.0% of patients had severe lymph node invasion (N3). The preceding data indicated that the majority of patients were diagnosed with late stages (III&IV). In terms of metastasis status, 25.0% of the patients developed metastatic dissemination to distant locations. Based on clinical examination of ovarian tumor markers, the mean of CA 125 was 733 U/ml and the HE4 mean was 494 pmol/L. All clinicopathological characteristics demonstrated that the higher stages are usually accompanied by more advanced disease and a less clinical outcome.

### Investigating the expression levels of ***KRAS*and*NOXA*** between ovarian cancer patients and controls

**Fig. 1:**
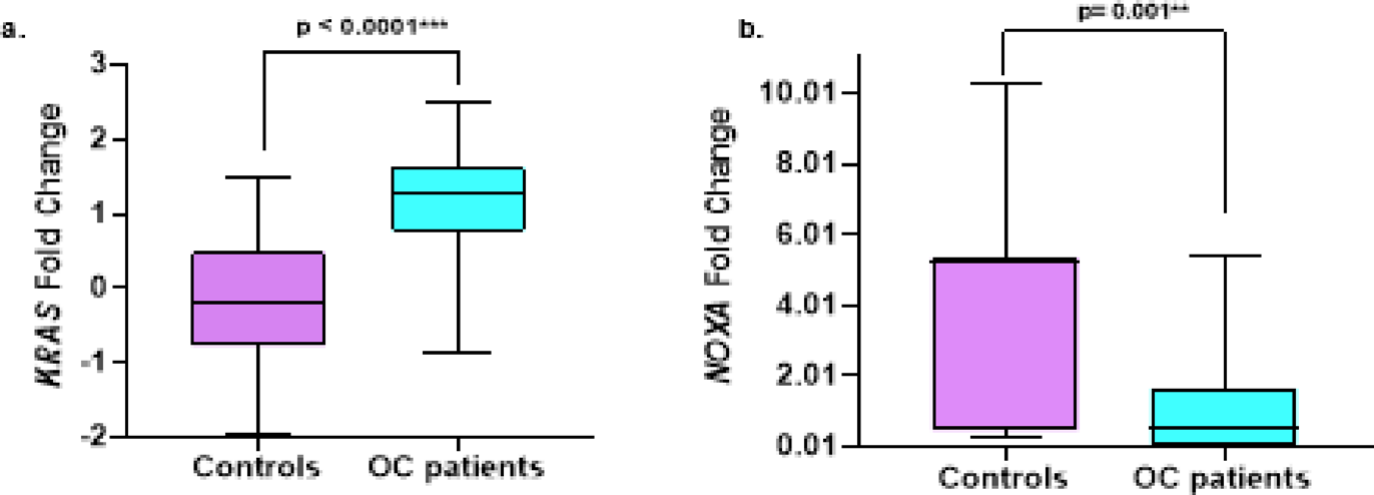
Gene Expression Profile of *KRAS* and *NOXA* in ovarian cancer patients (OC) and controls (**a**) Relative expression levels of *KRAS*, (**b**) Relative expression levels of *NOXA.*.

To explore the molecular involvement of *KRAS & NOXA* in ovarian cancer development, the level of *KRAS & NOXA* has been assessed in OC patients compared to controls. Our findings revealed that the expression level of *KRAS* was significantly upregulated in ovarian tumors compared to the control group (*p* < 0.0001) (Fig. 1a). However, the expression of *NOXA* was downregulated in ovarian cancer patients compared to the controls (*p* = 0.001) (Fig. 1b).

### Validation of ***KRAS*** and ***NOXA*** mRNA expression levels based on patients’ clinicopathological characteristics

**Fig. 2:**
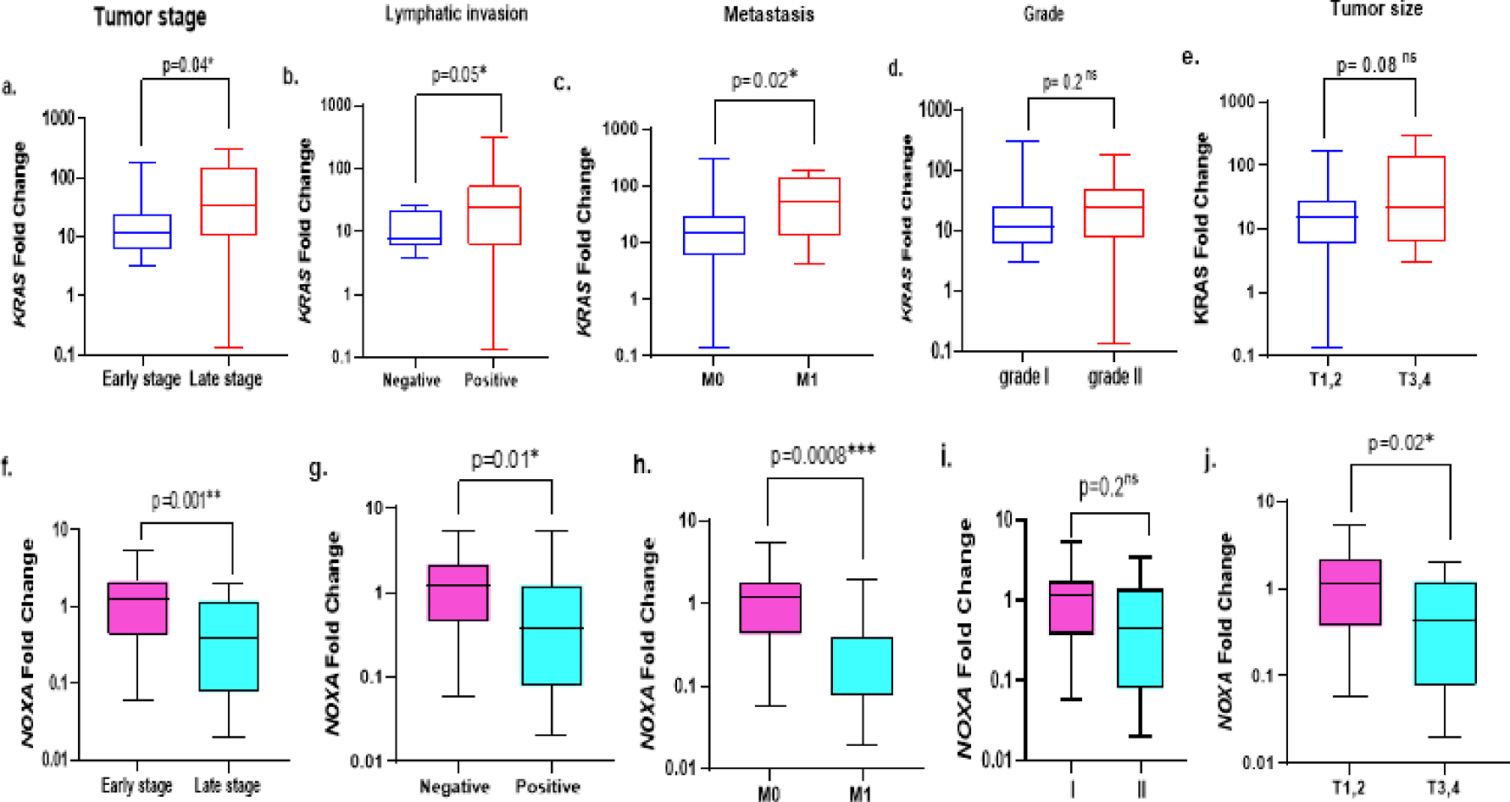
Relative gene expression levels of studied genes according to clinicopathological features. (**a**–**e**) represent *KRAS* gene expression, (**f**–**j**) represent *NOXA* gene expression.

To assess the impact of *KRAS* and *NOXA* on the clinicopathological severity of ovarian cancer patients, their expression levels were correlated and demonstrated in Fig. 2. The obtained data revealed that ovarian cancer patients with more aggressive clinicopathological data, including (advanced stage (III&IV), grade (II), lymph node invasion, big tumor size (T3&T4), and metastatic status (M1), had a remarkably elevation in *KRAS* expression level. Overexpression of *KRAS* achieved statistical significance with advanced stage, positive lymph invasion, and metastatic status as follows (*p* = 0.04, 0.05, 0.02, respectively) when compared to less aggressive features (early stage, negative lymph invasion, and undetected metastasis, respectively). Moreover, a noted trend in *KRAS* up-regulation was observed in grade (II) and big tumor size, but without showing any significance (*p* = 0.2, 0.07, respectively) (Fig. 2a-e). In contrast, the reduction in *NOXA* expression level was significantly correlated with the development of tumors with more aggressive features (*p* = 0.001, 0.01, 0.0008, 0.02, respectively) as illustrated in (Fig. 2f-j).

### Comparative analysis of ***KRAS*** and ***NOXA*** protein levels among studied groups

**Fig. 3:**
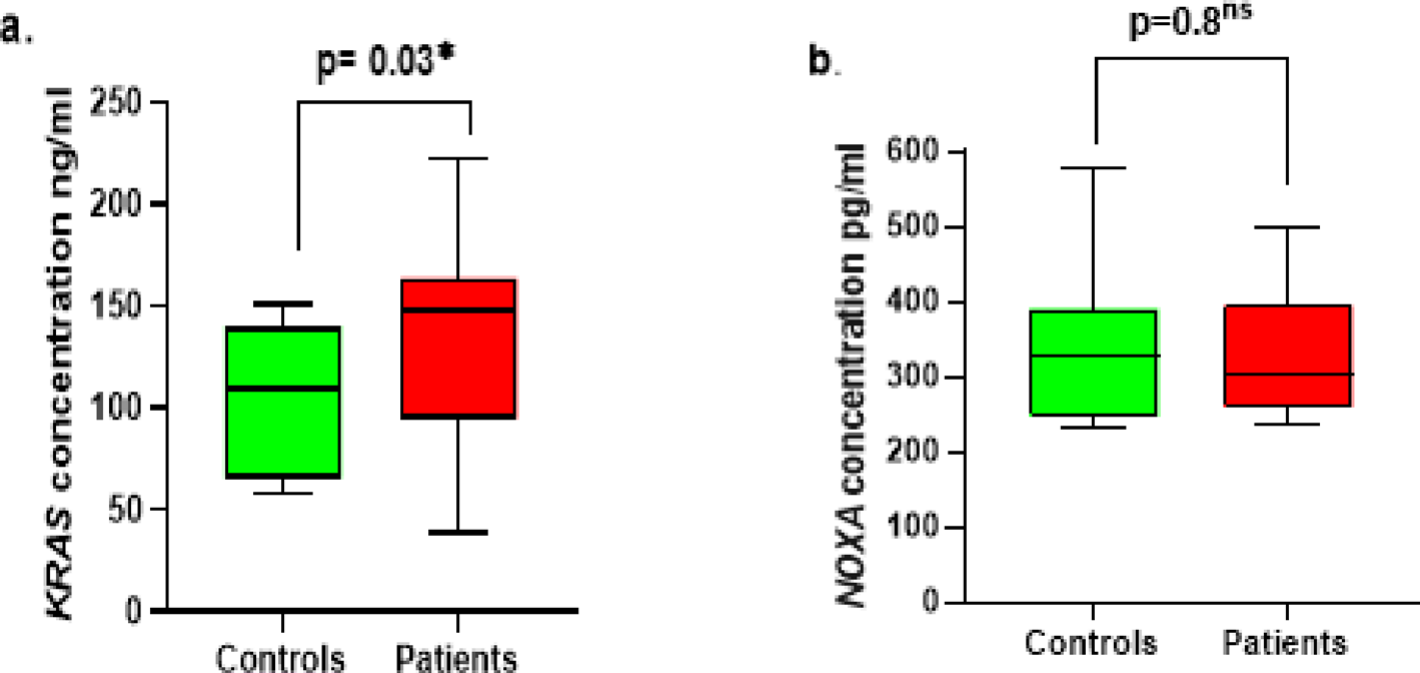
Serum concentration of *KRAS* and *NOXA* among cohort participants.

The serum concentrations of *KRAS* and *NOXA* were analyzed in both healthy individuals and patients with ovarian cancer (Fig. 3). It was shown that the serum protein level has been elevated significantly in OC patients compared to healthy controls (*p* = 0.03) (Fig. 3a). This finding indicated a potential association between the elevated level of *KRAS* and the occurrence of OC. On the other hand, the serum concentration of *NOXA* protein was found to be decreased in ovarian cancer patients relative to the control group. Nonetheless, this reduction did not reach statistical significance (*p* = 0.8) (Fig. 3b).

### Correlation between ***KRAS*** and ***NOXA*** protein levels in patients with various aspects of tumor aggressiveness

**Fig. 4:**
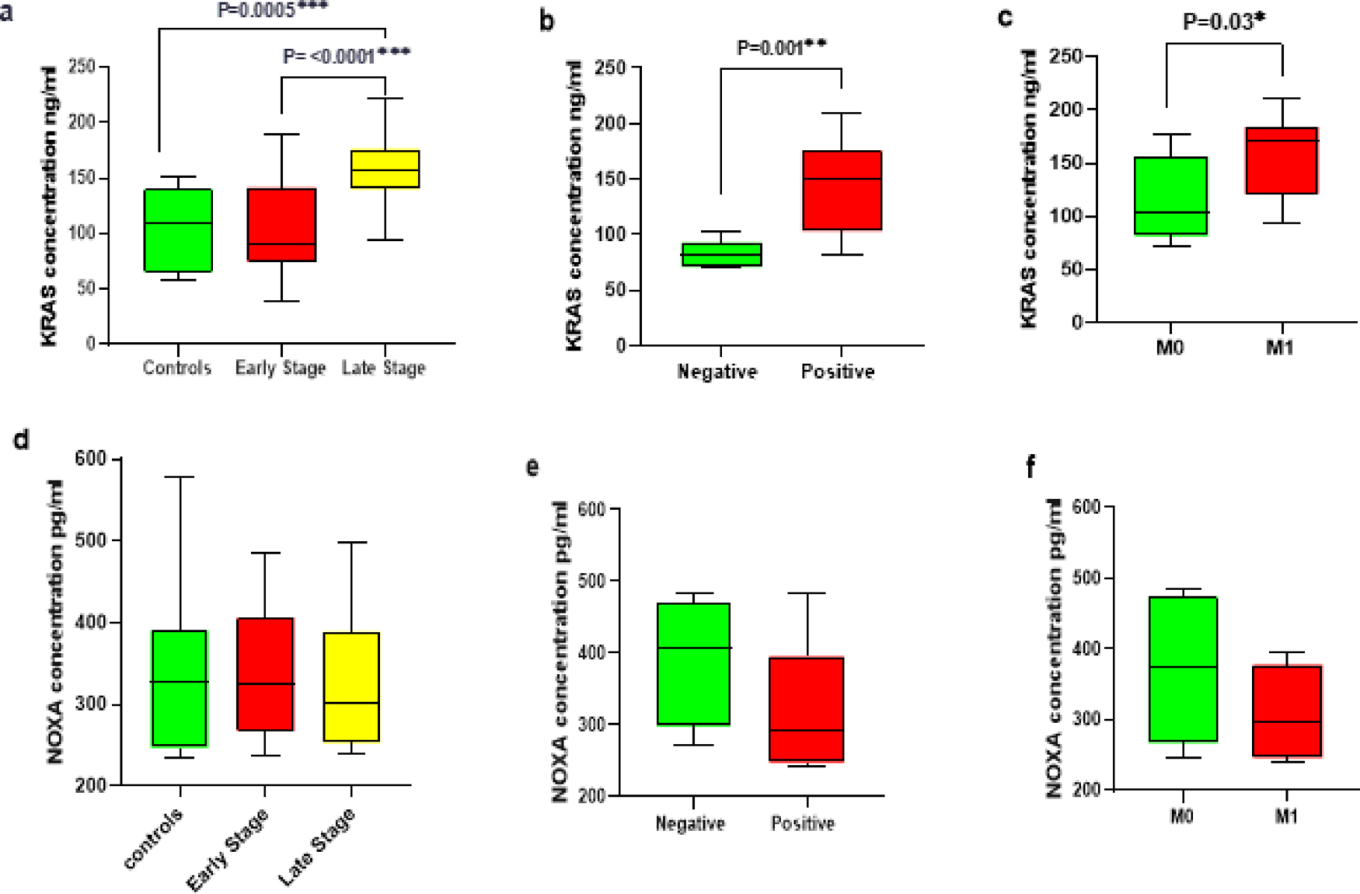
Analysis of the protein level for the studied genes in ovarian cancer patients. (**a**), (**b**), (**c**) *KRAS* protein levels in studied cohort according to tumor stage, lymph invasion and metastasis, respectively. (**d**), (**e**), (**f**) *NOXA* protein levels in studied cohort according to tumor stage, lymph invasion and metastasis, respectively.Comparisons were considered statistically significant at a level of *p* ≤ 0.05.

The correlation between the protein levels of *KRAS* and *NOXA* with tumor aggressiveness features has been assessed to explore their impact on ovarian cancer progression (Fig. 4). Ovarian tumors with advanced stages, positive lymph invasion, and metastatic status were classified as more aggressive group. Our findings revealed the presence of elevated levels of *KRAS* serum protein in patients with late-stage disease, lymphatic invasion, and metastasis to other organs. Notably, a significant statistical association between the levels of *KRAS* protein and the aggressive characteristics of the tumors was observed (*p* = < 0.0001, 0.001, and 0.03, respectively) (Fig. 4a, b, c). In contrast, we recorded a trend towards decreased levels of *NOXA* serum protein in patients with more aggressive forms of ovarian tumors; however, this decrease did not achieve statistical significance (*p* = 0.9, 0.2, 0.2, respectively) (Fig. 4d, e, f). Consequently, further investigation is warranted for *NOXA* in this context.

### The predictive ability of ***KRAS*** gene and protein as a tumor marker in ovarian cancer

**Fig. 5:**
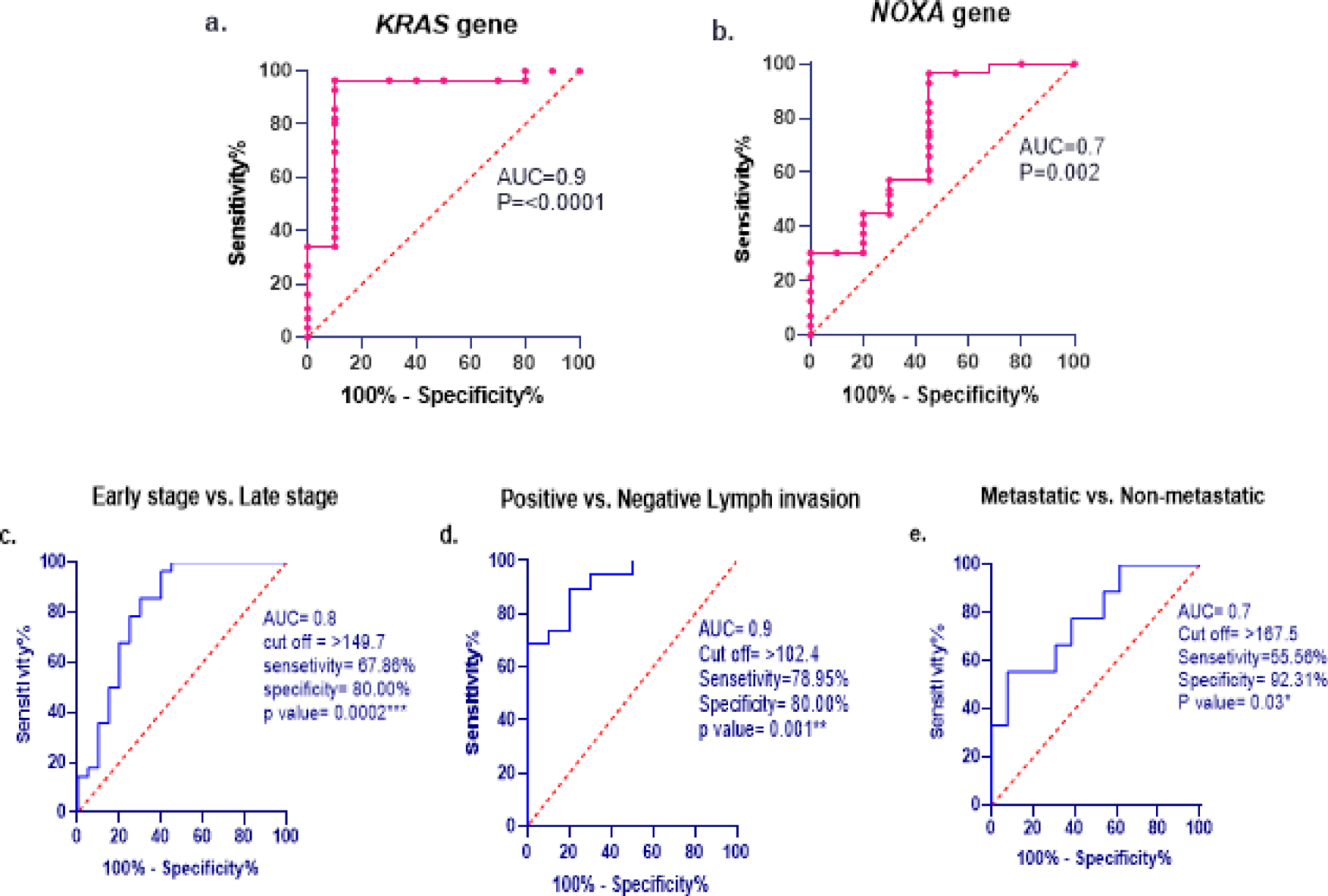
ROC curve analysis of *KRAS* and *NOXA* (**a**, **b**) ROC curve of *KRAS* and *NOXA* gene in malignant ovarian patients compared to controls, (**c–e**) demonstrate the diagnostic ability of *KRAS* protein in distinguishing between different aspects of ovarian cancer. (**c**) Progressive stage (early stage I, II & late stage III&IV), (**d**) Lymph invasion (positive & negative), (**e**) Metastaic status (M0 & M1).

The effectiveness of *KRAS* and *NOXA* as diagnostic biomarkers at the genetic level was assessed for their diagnostic ability to distinguish between ovarian cancer patients and the healthy control group (Fig. 5). The ROC curve data of the *KRAS* gene showed an AUC value of 0.9 with a cutoff > 3.067, sensitivity of 96.43%, and specificity of 90.00% (Fig. 5a). Regarding the *NOXA* gene, AUC was 0.7 with a cutoff value of < 2.794, sensitivity of 92.86%, and specificity of 55.00% (Fig. 5b).

Moreover, the *KRAS* proteomic level was correlated to various aspects of tumor progression through ROC analysis, as illustrated in Fig. 5 (c–e). The analysis revealed that the *KRAS* protein could be utilized as a promising discriminator in the differential diagnosis of ovarian cancer progression characteristics, including progressive stage, lymph node invasion, and metastatic status. The results indicated that serum *KRAS* exhibits a diagnostic accuracy in distinguishing between early and late stage of ovarian cancer (AUC = 0.8, 95% CI = 0.682 to 0.949, *P* = 0.0002), demonstrating a sensitivity of 87.86% and specificity of 80% at a cutoff > 149.7 ng/ml (Fig. 5c). Furthermore, ROC analysis demonstrated significant diagnostic power in differentiating between positive and negative lymphatic invasion (AUC = 0.9, 95% CI = 0.7994 to 1.000, *P* = 0.001), with a sensitivity of 78.95% and specificity of 80% at a cutoff of > 102.4 ng/ml (Fig. 5d). Additionally, *KRAS* can distinguish effectively between non-metastatic (M0) and metastatic status (M1), achieving a sensitivity of 55.56% and specificity of 92.31% at a cutoff > 167.5 ng/ml (AUC = 0.7, 95% CI = 0.580 to 0.975, *P* = 0.03) (Fig. 5e).

## Discussion

Ovarian carcinoma (OC) represents one of the leading causes of gynecological cancer related deaths with a poor prognosis. Ovarian cancer development and progression is a multistage process, which involves intricate molecular interactions^24^. To date, the molecular mechanisms that govern essential cellular processes such as cell growth, division, differentiation, and apoptosis in ovarian cancer are still not fully understood. Consequently, the ongoing research in oncology paves the way for the identification of genetic profile of cancer that helps in tumor monitoring. Therefore, the discovery of new molecular targets remains a significant clinical challenge in the medical era. The current study adopts the selection of a peripheral blood sampling strategy, rather than tissue biopsy, as it represents a less invasive technique that allows early detection and dynamic disease tracking, particularly when repeated tissue collection is not feasible^25^.

Ovarian cancer is often referred to as a “silent killer cancer” due to its subtle and nonspecific symptoms such as bloating, abdominal pain, and changes in appetite. The OC is usually diagnosed at an advanced stage, making treatment more difficult. The aggressive nature of OC allows it to spread quickly to other organs, complicating diagnosis and treatment. The early detection significantly improves survival rate^26^.

Age stratification of participants revealed that ovarian carcinoma was commonly diagnosed at age over 45 years old. This finding aligned with Jumma et al., study, which reported that 46.42% of ovarian cancer patients were older than 45 years old^27^. Furthermore, women over 50 years old have the highest incidence of ovarian cancer, with about 12.1% of cases occurring in those under 44 years old^28^.

With respect to menopause, the current data showed that ovarian cancer (OC) was most commonly diagnosed in postmenopausal women; 62.5% of epithelial ovarian cancer (EOC) patients were postmenopausal, compared to 37.5% who were premenopausal. This difference is statistically significant (*p* = 0.01). This finding was consistent with a multicenter case-control study indicating that 73% of EOC cases occurred after menopause among African-American women^29^.

The association between menopause and ovarian cancer risk was explored by different hypotheses. In menopause, the ovarian tissues undergo morphological transformation, known as “ovarian aging” which is thought to be the histological precursor of ovarian cancer. Furthermore, the cessation of ovarian hormone production during menopause leads to increased levels of gonadotropins, which may stimulate ovarian epithelial cells and contribute to tumorigenesis. The hormonal disruption is thought to be a risk factor for developing OC in postmenopausal women^30^.

It was shown that the CA125 is not a reliable biomarker for detecting ovarian cancer, particularly in premenopausal women, since its level can be elevated in muliple benign disorders such menstruation, pregnancy, and pelvic inflammatory illness, which result in numerous false positives. Additionally, CA125 lacks specificity because it can be elevated in other malignancies like breast, pancreatic, gastrointestinal, and lung cancers. Therefore, identifying transcriptomic and/or proteomic diagnostic biomarkers is essential to improve both accuracy and early detection of the OC^31^.

In recent years, the assessment of mRNA has gained significant interest in identifying and developing novel molecular biomarkers for the diagnosis and prognosis of various diseases, including cancer^32,33^. The identification of new biomarkers can aid in early detection and enhance patient prognosis. The discovery of non-invasive molecular markers is crucial for developing personalized medicine.

Interestingly, the *KRAS* and *NOXA* genes have gained significant attention in cancer research due to their crucial roles in tumor metabolism and apoptosis regulation, respectively, as both of them are fundamental to tumor development and progression. The *KRAS* gene drives metabolic alterations that support tumor growth, while *NOXA* regulates the balance between cell death and survival^23,34^.

In the current research, the mRNA levels of the *KRAS* and *NOXA* genes were assessed in the studied cohort. Regarding *KRAS*, the obtained data showed a high expression level of *KRAS* in the PBMCs of OC patients as compared to the control group (*p* < 0.0001). These findings are in accordance with the study by Yang et al., who further explored messenger RNA level of *KRAS* in different cancer types using the Oncomine database and found that *KRAS* was highly expressed in breast cancer, kidney cancer, lung cancer, myeloma, ovarian cancer, pancreatic cancer, and sarcoma compared to normal samples^18^. In addition, Zhou et al. demonstrated that *KRAS* overexpression was common in acute myeloid leukemia patients and was associated with low overall survival^35^.

*KRAS* activity is tightly regulated by the binding of GTP (active state) and GDP (inactive state). In normal cells, extracellular signals trigger GDP-to-GTP exchange, activating *KRAS* and downstream pathways such as RAL, RAF-MEK-ERK (MAPK/ERK), and PI3K-AKT, which govern cell proliferation and differentiation. After that, GTPase-activating proteins (GAPs) terminate signaling by hydrolyzing GTP to GDP^14^.

To shed more light on the potential role of the *KRAS* gene expression as a prognostic marker in ovarian cancer, the association between the expression level of *KRAS* gene and several clinicopathological parameters has been examined. The results revealed that there was a significant relationship between the upregulation of the *KRAS* gene and advanced clinical stages of OC, as well as an increased incidence of both lymphatic and distant metastasis ( *p* = 0.04, 0.05, 0.02, respectively), suggesting a potential role of *KRAS* in tumor progression and metastatic behavior. Our findings are in agreement with the data collected from 33 tumor expression datasets and genome set enrichment analysis (GSEA), which concluded that high *KRAS* expression levels have been observed among advanced clinical stages of various cancer types^36^. A recent supportive study on Oral Squamous Cell Carcinoma (OSCC) postulated that increased *KRAS* levels were linked to poorly differentiated tumors, more advanced pathological stages, and lymph invasion, suggesting the crucial role of KRAS in triggering both local and distant metastasis in OSCC^37^. On the other hand, there was no discernible relationship between the *KRAS* mRNA expression and TNM stage, histological tumor grade in non–small-cell lung cancer (NSCLC)^38^.

To further validate the observed upregulation of the *KRAS* gene at the transcriptomic level. The obtained data from proteomic analysis showed a statistically significant increase in *KRAS* protein in ovarian cancer patients as well as in patients with more aggressive tumor features. The concordance between the transcriptomic and proteomic levels of *KRAS* gene strengthens the validity of our findings and highlights the potential utility of the *KRAS* as a biomarker in more aggressive OC tumors.

In accordance with earlier literature, which noted that *KRAS* protein levels were higher in ovarian patients than in controls^23^. Another study found that 83% of KRAS protein was expressed in metastatic colon cancer-diabetic patients compared with 22% in colon cancer-nondiabetic patients^39^. Zhou et al. also documented the role of *KRAS* protein levels in cancer progression, as they were significantly correlated with higher rates of detected positive lymph nodes and venous invasion in CRC. Moreover, high *KRAS* protein levels were linked to worse overall survival and disease-free survival in CRC patients. These findings suggested that *KRAS* may serve as a prognostic marker for evaluating cancer outcomes^40^.

These results were confirmed by performing ROC curve analysis which demonstrated that *KRAS* gene expression level exhibited excellent diagnostic value in distinguishing ovarian cancer patients from healthy controls (AUC 0.9,* p* = < 0.001). Moreover, its transcriptomic level was a good discriminator in disease progression that included advanced stages and dissemination of malignant tumor to the lymphatic system and other organs in the body (*p* = 0.0002, 0.001, 0.03, respectively), and it achieved considerable AUC values (0.9, 0.8, 0.7, respectively). These data highlighted the genetic and transcriptomic levels of *KRAS* as powerful diagnostic biomarkers in prognosis and predicting ovarian cancer progression.

Concerning *NOXA* expression, its expression level was evaluated in PMBCs of OC patients compared to healthy individuals. There was a significant reduction in *NOXA* expression level (*p* = 0.001). These results were consistent with findings that have highlighted a significant downregulation of *NOXA* in human adenoid cystic carcinoma (ACC) and gastric cancer (GC). Furthermore, enhancing *NOXA* expression inhibits the growth and invasion of both cancers^22,41^. Accumulating evidence from cancer therapy research suggested that resistance to therapy was frequently correlated with the downregulation of *NOXA* mRNA in various cancer types^42,43^. The ROC curve results confirmed the diagnostic utility of the *NOXA* gene to differentiate between patients and controls (AUC 0.7, *p* = 0.002). These findings align with the hypothesis that functional *NOXA* plays a pivotal role in the execution of effective apoptosis in response to therapeutic interventions.

*NOXA* plays a critical role in modulating cancer initiation and progression through intricate molecular and cellular pathways. The expression of *NOXA* can be upregulated in response to DNA damage and oncogenic signals, leading to a genetic repair mechanism and ultimately preventing the proliferation of cancerous cells. Additionally, *NOXA* orchestrates the intrinsic apoptotic pathways by interacting with other members of the Bcl-2 family. Furthermore, *NOXA* helps in the autophagy breakdown of damaged organelles, thereby promoting cellular survival. Therefore, the dysregulation of the *NOXA* gene plays a role in the development and progression of carcinoma, indicating that the *NOXA* gene could serve as a potential target marker for therapeutic strategies^21^.

Furthermore, the association between *NOXA* expression level and the clinicopathological criteria of the tumor has been studied. It was found that the downregulation of the *NOXA* gene was strongly associated with tumor aggressiveness features (advanced stages, lymph invasion, tumor size and distant metastasis) (*p* = 0.001, 0.01, 0.0008, 0.02, respectively) ). Our results were aligned with the findings of Liang et al., who demonstrated that the *NOXA* expression level was negatively correlated with the clinicopathological parameters of ACC^22^. However, the *NOXA* expression was not correlated with the stage of NSCLC^44^. It was reported that the downregulation of *NOXA* gene was linked to poor survival outcomes in ACC patients, indicating the prognostic value of the *NOXA* gene^22^.

The validation of transcriptomic data of the *NOXA* gene has been assessed by measuring its protein level. The *NOXA* protein level in the ovarian cancer patients was found to be lower than in the control group, although this difference did not achieve statistical significance (*p* = 0.8). However, olbromski et al. observed a significant decrease in the level of *NOXA* protein in ovarian cancer patients^23^. Moreover, a significant downregulation of *NOXA* protein level was observed in gastric cancer samples compared to adjacent normal tissues^41^.

Concerning clinicopathological parameters, it was observed that the protein level of *NOXA* was decreased with the severity features of ovarian tumors without reaching any significance (*p* = 0.9, 0.2, 0.2, respectively). The sample size of patients should be increased to investigate the *NOXA* correlation with clinicopathological parameters. These results aligned with Al Shboul et al., study which indicated the association between the increased expression levels of *NOXA* protein and the negative status of lymph nodes in breast cancer patients^45^. Furthermore, low *NOXA* expression was correlated with advanced T stage (T3,4) and distant metastasis in GC tissues. Additionally, gastric cancer patients with low expression of *NOXA* had a poorer prognosis. It’s worth noting that *NOXA* overexpression had an inhibitory effect on gastric cancer cell proliferation, invasion, and migration^41^. In conclusion, the current study revealed the oncogenic ability of the *KRAS* gene through promoting tumor growth and the tumor suppressor function of the *NOXA* gene through its apoptotic characteristics in ovarian cancer development and progression.

We acknowledge several potential limitations of this study. Firstly, the small sample size may limit the statistical robustness and hinder the validation of the obtained data. The study also does not include long-term follow-up data or survival analysis. Moreover, it would be interesting to investigate whether these markers identified in the serum of ovarian cancer patients demonstrate a similar pattern in tissue samples. These limitations underscore the necessity for further research involving larger cohorts with extensive clinical data and tissue-specific analysis before these findings can be translated into diagnostic and prognostic applications.

## Materials

### Sample size

The sample size was calculated using G*Power software 3.1.9.2 .

### Study participants

The current research involved 56 Egyptian women, aged between 27 and 73 years. Participants were selected from the gynaecology outpatient clinic during the period from December 2022 to May 2023. All cases were confirmed as having an ovarian tumour at the Clinical Pathology Department, National Cancer Institute by routine pathology and immunohistochemistry. All biological samples were collected at the time of diagnosis before the start of any treatment strategy. Patients with a known history of other malignant diseases, uncontrolled diabetes, renal impairment, and other serious concurrent illnesses were excluded. All demographic data, clinical examinations, and pathology reports were recorded. The enrolled OC patients were staged and graded using the International Federation of Gynaecology and Obstetrics (FIGO) clinicopathological staging, which categorized patients into four stages (I-IV) and three grades (I-III), based on the extent of cancer spread and tumor cell differentiation, respectively^46^.

The control group included twenty healthy age-matched women with no history of cancer, hypertension or diabetes mellitus. The study was authorized by the Institutional Review Board (IRB) of the National Cancer Institute Ethics Committee (No. CP0923-403-06). Written informed consent was obtained from each participant. All procedures followed Helsinki Declaration guidelines regarding medical research with human subjects.

### Extraction of RNA

The peripheral blood samples and sera were collected. The total cellular RNA was extracted from whole blood using the guanidinium thiocyanate-phenol-chloroform technique outlined by^47^. The extracted RNA was subjected to NanoDrop (UV–VIS-Spectrophotometer Q 5000, USA) to measure the purity and RNA concentration. The separated serum was used to assess the protein levels.

### Quantitative real‑time PCR

The relative expression of *KRAS* and *NOXA* mRNAs in all samples was evaluated using the QuantiNova SYBR Green RT-PCR Kit ( Cat.No 208152) (Qiagen GmbH, Germany). The reaction mixture included 250 ng of purified RNA, 10 µl 2x QuantiTect SYBR‑Green Master Mix, 0.2 µl QN SYBR Green RT-Mix, and 1 µl 20x QuantiTect IC Primer assay of the target gene. Finally, the reaction volume was adjusted to 20 µl using Q-water. The thermal profile was as follows: RT-step was 10 min at 50 °C followed by initial incubation for 2 min at 95 °C, 40 cycles at 95 °C for 5 s, and 60 °C for 10 s. Visualization and exportation of data were performed using the rotor-gene real-time PCR system (Qiagen GmbH). The expression levels of *KRAS* and *NOXA* genes were quantified by using the comparative threshold cycle method (2^−ΔΔCt). The data were normalized to the housekeeping gene Glyceraldehyde-3-phosphate dehydrogenase (*GAPDH*) (Qiagen Hilden, Germany), and the healthy control group was served as the calibrator for relative quantification.

### Measurement of ***KRAS & NOXA*** protein levels in serum

Serum *KRAS* and *PMAIP1/NOXA* levels were quantified by a sandwich enzyme-linked immunosorbent assay (ELISA) utilizing a human ELISA kit (Cat. No: E-EL-H2182) and (Cat. No: SL3667hu) respectively. All measurements were done according to the manufacturer’s instructions. Samples were performed in duplicates. The measurements were performed at 450 nm. The concentration of the protein in the serum was calculated from the generated standard curve using the standards provided with the kits.

### Statistical analysis

The data were analyzed using GraphPad Prism 8.0.0. Numerical data are presented as the mean ± standard deviation, and qualitative data are presented as numbers and percentages. Depending on the variable distribution, the one-way ANOVA or the Kruskal-Wallis test was used for multi-group comparison. Furthermore, the Student’s t-test or the Mann-Whitney U test was used for pairwise comparison. The receiver operating characteristic (ROC) curve was utilized to evaluate the ability of a molecular marker to differentiate between two populations. The sensitivity, specificity, and cut-off were computed. The criterion for statistical significance was established at *P* values ≤ 0.05.

## Data Availability

The data presented in this study are available in this article.
